# Enhanced TLR7-dependent production of type I interferon by pDCs underlies pandemic chilblains

**DOI:** 10.1084/jem.20231467

**Published:** 2025-04-14

**Authors:** Fanny Saidoune, Danyel Lee, Jeremy Di Domizio, Corentin Le Floc’h, Raphael Jenelten, Jérémie Le Pen, Vincent Bondet, Ana Joncic, Marie-Anne Morren, Vivien Béziat, Shen-Ying Zhang, Emmanuelle Jouanguy, Darragh Duffy, Charles M. Rice, Curdin Conrad, Jacques Fellay, Jean-Laurent Casanova, Michel Gilliet, Ahmad Yatim

**Affiliations:** 1 Department of Dermatology, CHUV University Hospital and University of Lausanne, Lausanne, Switzerland; 2 St. Giles Laboratory of Human Genetics of Infectious Diseases, Rockefeller Branch, The Rockefeller University, New York, NY, USA; 3 Laboratory of Human Genetics of Infectious Diseases, Necker Branch, INSERM U1163, Paris, France; 4 Imagine Institute, Paris Cité University, Paris, France; 5 Translational Immunology Unit, Institut Pasteur, Université Paris Cité, Paris, France; 6 School of Life Sciences, Ecole Polytechnique Fédérale de Lausanne, Lausanne, Switzerland; 7 Precision Medicine Unit, Lausanne University Hospital and University of Lausanne, Lausanne, Switzerland; 8 Department of Pediatrics, Necker Hospital for Sick Children, Paris, France; 9 Howard Hughes Medical Institute, New York, NY, USA; 10 Laboratory of Virology and Infectious Disease, The Rockefeller University, New York, NY, USA

## Abstract

Outbreaks of chilblains were reported during the COVID-19 pandemic. Given the essential role of type I interferon (I-IFN) in protective immunity against SARS-CoV-2 and the association of chilblains with inherited type I interferonopathies, we hypothesized that excessive I-IFN responses to SARS-CoV-2 might underlie the occurrence of chilblains in this context. We identified a transient I-IFN signature in chilblain lesions, accompanied by an acral infiltration of activated plasmacytoid dendritic cells (pDCs). Patients with chilblains were otherwise asymptomatic or had mild disease without seroconversion. Their leukocytes produced abnormally high levels of I-IFN upon TLR7 stimulation with agonists or ssRNA viruses—particularly SARS-CoV-2—but not with DNA agonists of TLR9 or the dsDNA virus HSV-1. Moreover, the patients’ pDCs displayed cell-intrinsic hyperresponsiveness to TLR7 stimulation regardless of TLR7 levels. Inherited TLR7 or I-IFN deficiency confers a predisposition to life-threatening COVID-19. Conversely, our findings suggest that enhanced TLR7 activity in predisposed individuals could confer innate, pDC-mediated, sterilizing immunity to SARS-CoV-2 infection, with I-IFN–driven chilblains as a trade-off.

## Teaser

Chilblains were reported in SARS-CoV-2–exposed individuals without PCR- or serologically defined COVID-19. Excessive TLR7 responses to SARS-CoV-2 by pDCs are linked to I-IFN–driven chilblain development. While TLR7 deficiency predisposes to severe COVID-19, enhanced TLR7 responses may confer sterilizing immunity to SARS-CoV-2, with chilblains as a trade-off.

## Graphical Abstract

**Figure:**
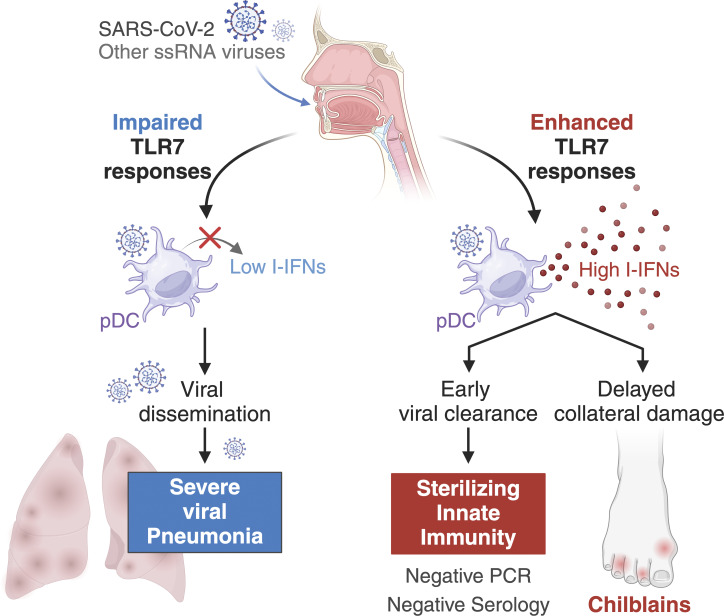

## Introduction

Chilblains manifest as red-to-violaceous papules and painful swellings at acral sites. This rare inflammatory condition, also known as pernio, can be primary (“idiopathic”) or secondary. Secondary forms are a characteristic manifestation of type I interferon (I-IFN)–driven diseases. Their strong association with monogenic type I interferonopathies, such as familial chilblain lupus, Aicardi–Goutières syndrome, and STING-associated vasculopathy with infantile onset (Crow and Stetson, 2022), and with systemic lupus erythematosus (Arora et al., 2024; Hutchinson, 1888), suggests that I-IFN is involved in their development. Primary forms, also known as seasonal chilblains, affect otherwise healthy individuals in the winter (Chan et al., 2008; Larkins and Murray, 2013; McCleskey et al., 2021). With an annual incidence rate of 5 per 100,000 individuals, seasonal chilblains were exceedingly rare before the COVID-19 pandemic (McCleskey et al., 2021). Like secondary forms, they are characterized by high levels of I-IFN activity (Arkin et al., 2024; De Greef et al., 2022; Frumholtz et al., 2021; Magro et al., 2021). During cold seasons, clusters of seasonal chilblain cases coincide with the periods of circulation of winter-specific single-stranded RNA (ssRNA) viruses, such as seasonal coronaviruses, respiratory syncytial virus, and influenza A (Moriyama et al., 2020). However, no direct link to viral triggers has been firmly established.

The COVID-19 pandemic provided a unique opportunity to investigate the potential role of viral infection in chilblain pathogenesis. COVID-19 outbreaks were accompanied by an unprecedented increase in the number of cases of primary chilblains (Freeman et al., 2020; McCleskey et al., 2021), often described as “COVID toes” or pandemic chilblains (PC) in this context. Unlike seasonal chilblains, which typically occur in late January in the Northern Hemisphere (McCleskey et al., 2021) and late June in the Southern Hemisphere (Larkins and Murray, 2013; Sawires et al., 2022), PC cases in this context coincided with local waves of COVID-19. The temporal clustering of cases of chilblains with cases of COVID-19 (Bascuas-Arribas et al., 2022; Giraud-Kerleroux et al., 2021; Moghadam et al., 2021), with the peak incidence of chilblains occurring 2 wk after the infection peak (Feito-Rodriguez et al., 2021), suggests a tight causal link between SARS-CoV-2 and chilblains.

Epidemiological studies confirmed a marked increase in chilblain cases during the pandemic that was significantly correlated with SARS-CoV-2 circulation (McCleskey et al., 2021; Sawires et al., 2022), and established household exposure to SARS-CoV-2 in cases of chilblains (Hubiche et al., 2021b; Poizeau et al., 2022). Evidence of abortive SARS-CoV-2 infection has also been reported in PC patients. Viral RNA debris, with no evidence of replication, was detected in about 25% of cases from two cohorts, one of which was our own cohort (Arkin et al., 2024). An early transient SARS-CoV-2–specific IgA response was observed in about 20–30% of PC cases in multiple studies (Bessis et al., 2022; El Hachem et al., 2020; Frumholtz et al., 2021; Hubiche et al., 2021a). However, the overall low rate of IgG seroconversion across studies (below 10%) and the absence of detectable viral replication at the onset of chilblains (observed in <5% of cases) have made it challenging to establish a definitive link between PC and SARS-CoV-2 infection (Baeck et al., 2021; Caselli et al., 2020; Cassius et al., 2021; Gehlhausen et al., 2022; Le Cleach et al., 2020; Lesort et al., 2020; Sánchez-García et al., 2022). Various hypotheses have been proposed, including a relationship to the sedentary lifestyle developed during lockdown (exposure of the feet to cold) (Gehlhausen et al., 2022; Herman et al., 2020; Neri et al., 2020) and rapid clearance of the virus by innate immunity, involving I-IFNs in particular (Arkin et al., 2021; Frumholtz et al., 2021; Hubiche et al., 2021a; Lipsker, 2020; Mensa-Vilaró et al., 2022; Yatim and Gilliet, 2021). Despite the considerable efforts made, the reasons for the outbreaks of chilblains during the pandemic have remained unclear.

Impaired I-IFN immunity predisposes individuals to life-threatening viral infections (Casanova and Abel, 2022), including critical COVID-19 pneumonia (Asano et al., 2021; Bastard et al., 2021; Bastard et al., 2020; Zhang et al., 2020). Conversely, excessive I-IFN activity can be harmful and drives numerous autoinflammatory and autoimmune diseases (Brown et al., 2022; David et al., 2024; Tusseau et al., 2024). Thus, human inborn errors underlying insufficient or excessive I-IFN levels highlight the narrow window of activity over which I-IFNs have beneficial effects (Crow and Casanova, 2024). We hypothesized that individuals with PC are predisposed to the mounting of enhanced I-IFN responses to SARS-CoV-2, promoting early viral clearance and manifesting clinically as chilblains.

## Results and discussion

### Primary chilblains during COVID-19 outbreaks

**Figure 1.**
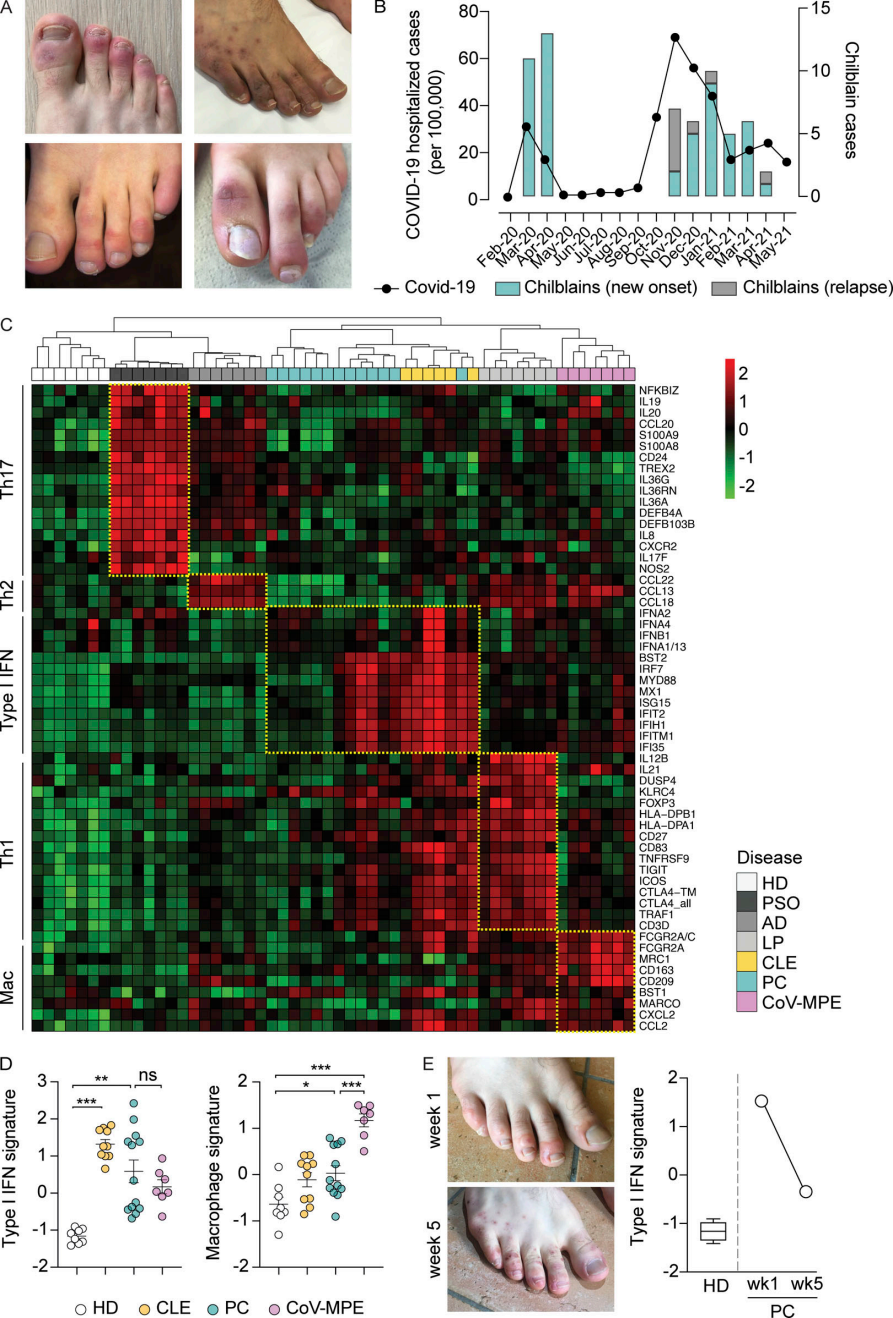
**Dominant I-IFN signature in PC. (A)** Photographs of PC (COVID toes). **(B)** Temporal association between chilblain outbreaks and local waves of COVID-19. The line shows the number of hospitalizations for laboratory-confirmed COVID-19 in Switzerland (admissions per month per 100,000 inhabitants) (Bundesamt für Statistik, 2022). The histograms show the number of chilblain cases enrolled per month (initial onset and relapses). **(C)** NanoString profiling of immune gene expression for PC and common inflammatory skin diseases. The heatmap shows Z-scores with unsupervised clustering of immune gene expression profiling results for PC (*n* = 13), plaque-type psoriasis (PSO, *n* = 7), atopic dermatitis (AD, *n* = 7), lichen planus (LP, *n* = 7), CLE (*n* = 6), CoV-MPE (*n* = 7), and healthy skin (HD, *n* = 7). Differentially expressed genes were used to generate disease-related gene signatures, including T helper (Th)17, Th2, Th1, I-IFN, and macrophage (Mac) signatures (immune modules). Red corresponds to upregulated genes, and green to downregulated genes. The yellow dashed lines indicate the upregulated immune modules across patient groups. **(D)** Type I IFN signature (left) and macrophage signature (right) in HD and inflammatory lesions from individuals with CLE, PC, and CoV-MPE. Bars represent the mean and SEM. **(E)** Photographs of lesions taken from the same patient during the early inflammatory phase (week 1, wk1) and the postinflammatory phase (week 5, wk5). Right panel: I-IFN signature in skin biopsy specimens from the lesions shown in the left panel, and for healthy skin from volunteers (HD, *n* = 7). The scores in D and E were calculated by determining the normalized mean expression level of the corresponding genes across all samples (Z-score). The adjusted P values in D were obtained in Brown–Forsythe Welch’s ANOVA followed by Dunnett’s test for multiple comparisons for datasets that were normally distributed, and Kruskal–Wallis tests followed by Dunn’s test for multiple comparisons for datasets that were not normally distributed. ***P < 0.001; **P < 0.01; *P < 0.05; ns = nonsignificant.

During the initial waves of the COVID-19 pandemic in Switzerland, between March 2020 and April 2021, we enrolled a cohort of 57 patients (Table S1) presenting chilblain lesions (Fig. 1 A). After the exclusion of alternative diagnoses and secondary causes (lupus erythematosus, I-IFN interferonopathies, and related autoinflammatory or autoimmune diseases), the lesions were classified as PC, the primary form occurring during the waves of the COVID-19 pandemic. The 57 patients included 38 women (67%), and age at onset ranged from 9 to 69 years (median of 32 years) (Table S1). The median duration of the lesions was 6 wk (Table S1). We observed a clear temporal association between the numbers of cases of chilblains seen at the clinic during the pandemic waves and the number of cases of COVID-19 reported in the population (Fig. 1 B). Patients with PC reported close contact with infected individuals, usually 2–4 wk before the onset of the chilblain lesions. More than half the cases of chilblains (32 of 56) occurred following contact with an individual with suspected or confirmed COVID-19 (Table S1). Despite suspected SARS-CoV-2 exposure, only 33% of chilblain patients (17 of 52) reported mild, nonspecific symptoms (Table S1), the majority of patients remaining asymptomatic. None of the patients with PC developed severe viral disease. In addition, nasopharyngeal swabs typically tested negative for SARS-CoV-2 mRNA (34 of 36 tested, 94%), indicating the absence of mucosal viral replication at the onset of chilblains (Table S1). Furthermore, serological tests failed to detect SARS-CoV-2–specific IgG in most chilblain patients (44 of 50 tested, 88%), with such antibodies detected in only six of the patients tested (12%) (Table S1). These observations are consistent with previous studies, indicating a low rate of seroconversion in individuals with PC.

### Dominant I-IFN signature in PC

We investigated the immunological mechanisms at work in PC, by performing a transcriptomics analysis on skin lesions from 13 patients. The results were compared with those for patients with other inflammatory skin diseases, including psoriasis, atopic dermatitis, lichen planus, cutaneous lupus erythematosus (CLE), and maculopapular skin rashes associated with moderate-to-severe COVID-19 (COVID-19–associated maculo-papular eruption [CoV-MPE]). Our transcriptomics analysis revealed a characteristic I-IFN signature in PC, in which the expression of I-IFN genes (*IFNA2*, *IFNA4*, *IFNA1/13*, and *IFNB1*) and IFN-stimulated genes (ISGs) (*BST2*, *IRF7*, *MX1*, *ISG15*, *IFIT2*, *IFIH1*, *IFITM1*, *IFI35*) was upregulated (Fig. 1 C). This pattern of gene expression closely mirrored that observed in CLE but not in the other dermatologic conditions considered (Fig. 1 C). The I-IFN signature was strongly induced in 7 of the 13 chilblain patients tested, whereas a weaker IFN signature was detected in the remaining six cases, possibly reflecting the transient nature of the inflammatory response (Fig. 1, C and D). Indeed, the inflammatory signature was most prominent in samples collected within 2 mo of the onset of chilblains (7 of 10 samples) and was weaker in samples obtained beyond this time point (3 samples) (Table S1). A comparison of biopsy samples taken from the same patient during the early inflammatory phase (red, swollen lesion) and the postinflammatory phase (purple lesion), 1 and 5 wk after onset, respectively, revealed a markedly weaker I-IFN signature in the later sample (Fig. 1 E). The IFN response is therefore, transient, occurring early in the course of chilblains.

### Large numbers of activated plasmacytoid dendritic cells (pDCs) in PC

**Figure 2.**
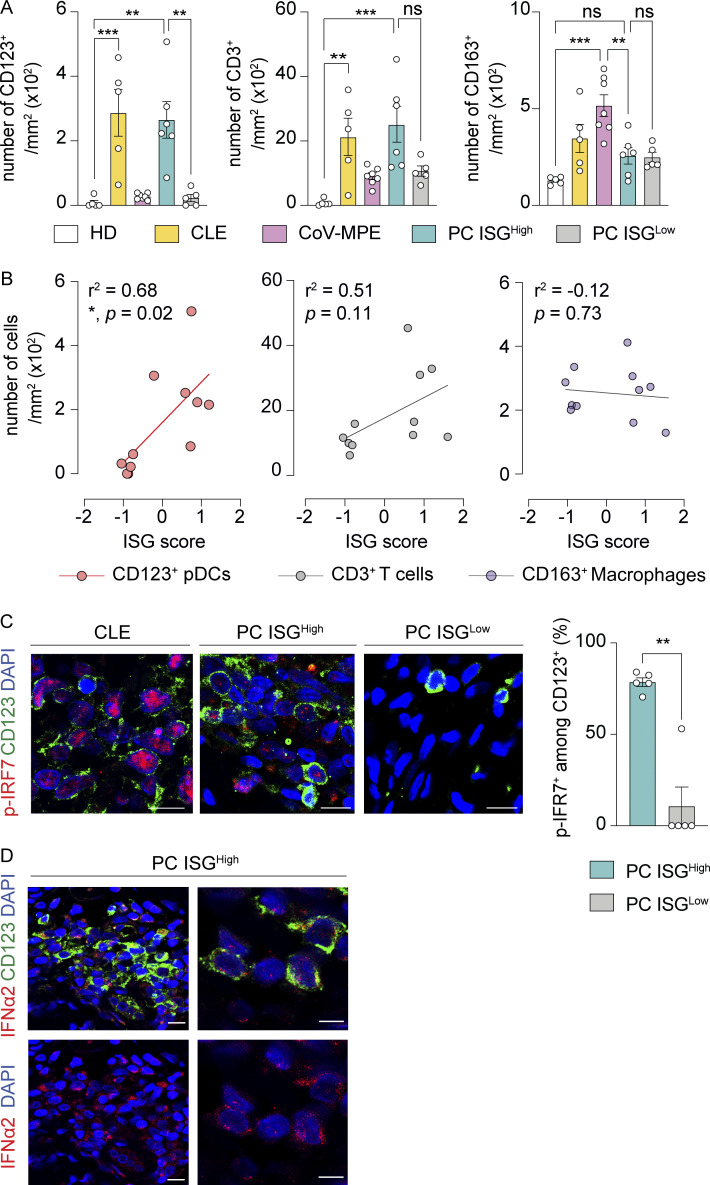
**Large numbers of pDCs in PC. (A)** Quantification of CD123^+^ cells (marker of pDCs), CD3^+^ cells (T cells), and CD163^+^ cells (macrophages) in healthy skin (HD, *n* = 5), CLE (*n* = 5), CoV-MPE (*n* = 7), and PC with a high (PC ISG^High^, *n* = 6) or low I-IFN signature score (PC ISG^Low^, *n* = 5). Adjusted P values were obtained in one-way ANOVA followed by Tukey’s tests for multiple comparisons. **(B)** Correlation between the ISG signature score calculated from the mean level of expression of *IRF7*, *MYD88*, *MX1*, *ISG15*, *IFITM1*, and *IFI35* (normalized across samples) and the number of CD123^+^ pDCs, CD3^+^ T cells, and CD163^+^ macrophages in PC lesions. Correlations were analyzed in Pearson’s tests. **(C)** Confocal microscopy images of representative CLE, ISG^High^ PC, and ISG^Low^ PC skin lesions stained for CD123 (green) and phosphorylated-IRF7 (p-IRF7, red). Scale bar, 10 μm. Right panel: proportions of CD123^+^ pDCs displaying nuclear p-IRF7^+^ staining in ISG^high^ PC and ISG^Low^ PC. Adjusted P values were obtained in Mann–Whitney tests. **(D)** Confocal microscopy images of ISG^high^ PC lesions stained for CD123 (green) and IFN-α2 (red). Left panel: low magnification. Scale bar, 10 μm. Right panel: high magnification. Scale bar, 5 μm. ***P < 0.001; **P < 0.01; *P < 0.05; ns = nonsignificant.

**Figure S1.**
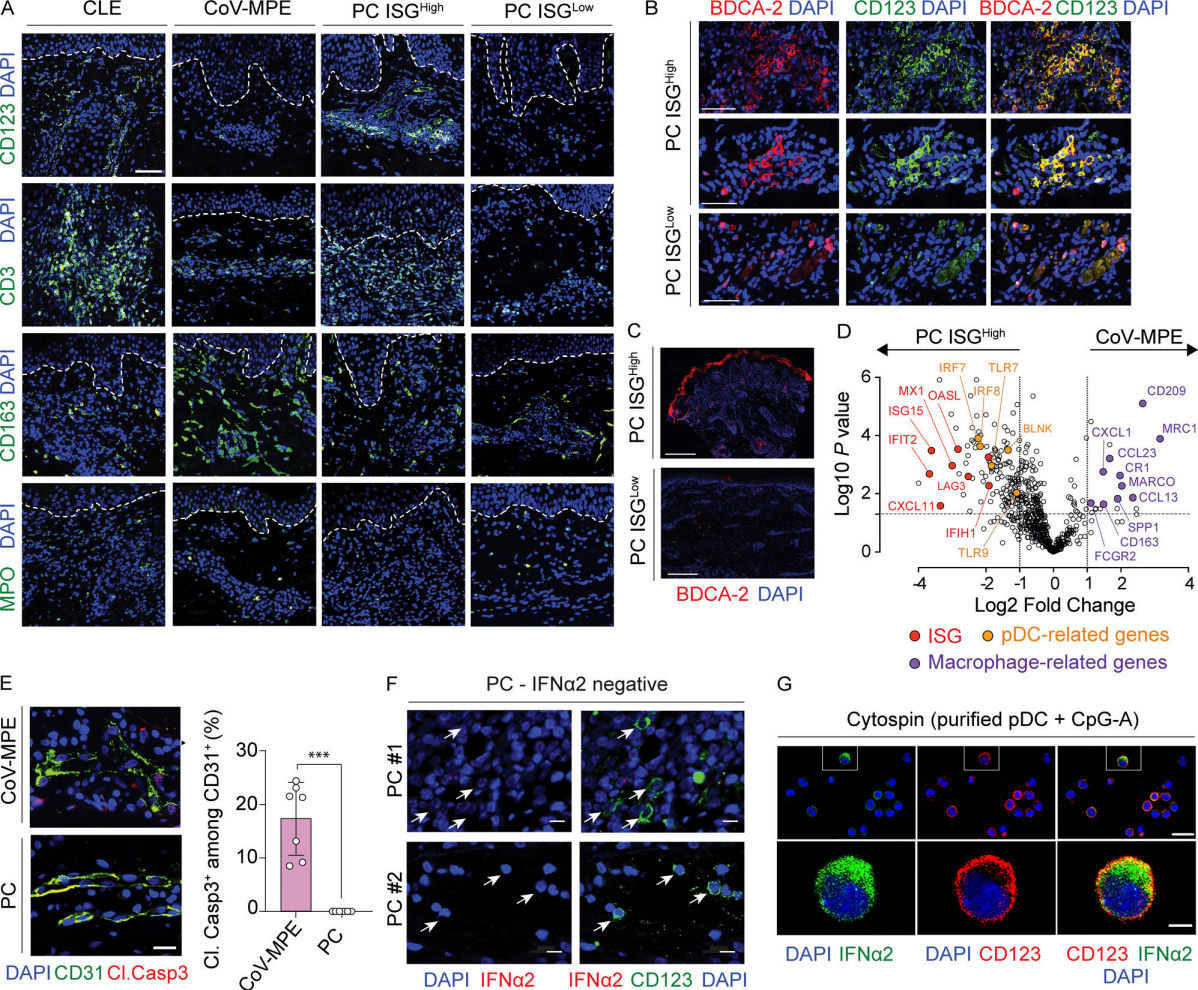
**Related to** Fig. 2. **(A)** Confocal microscopy images of CD123^+^ cells (marker of pDCs), CD3^+^ cells (T cells), CD163^+^ cells (macrophages), and MPO^+^ cells (neutrophils) in skin lesions from individuals with CLE, CoV-MPE, and ISG^high^ and ISG^Low^ PC. Representative images from 5 CLE, 7 CoV-MPE, 6 PC ISG^high^, and 5 PC ISG^Low^ patients are shown. Scale bars, 50 μm. **(B)** Confocal microscopy images of representative PC ISG^high^ and PC ISG^Low^ skin lesions stained for CD123 (green) and BDCA-2 (red). Scale bar, 50 μm. **(C)** Microscopy images of representative PC ISG^high^ and PC ISG^Low^ skin lesions stained for BDCA-2 (red). Scale bar, 500 μm. **(D)** Volcano plot of differentially expressed genes (DEG) in CoV-MPE (right) versus PC ISG^high^ (left). Macrophage-related DEG (purple), pDC-related DEG (orange), and type I ISG (red) are highlighted. **(E)** Absence of endothelial cell death in PC. Confocal microscopy images (left) and quantification (right) of cleaved caspase-3 (Cl.Casp3) in CD31^+^ endothelial cells in skin lesions from CoV-MPE (*n* = 7) and PC patients (*n* = 6). Scale bar, 20 μm. ***P < 0.001. **(F)** Negative controls for IFN-α2 staining. Confocal microscopy images of representative sections from two IFN-α2–negative PC skin lesions stained for CD123 (green) and IFN-α2 (red). Each skin sample is displayed in either the upper or the lower panel (scale bar, 10 μm). Arrows indicate CD123^+^ pDC cells. **(G)** Positive controls for IFN-α2 staining. Confocal microscopy images of cytospin preparations stained for IFN-α2 (green) and CD123 (red). pDC-enriched fractions from fresh PBMCs were stimulated with CpG-A prior to cytospin. The upper row shows images at low magnification (scale bar, 20 μm), and the lower row shows images at high magnification (scale bar, 5 μm).

We then investigated the immunological and cellular mechanisms underlying I-IFN production in PC. We performed immunofluorescence-based quantifications of the immune cells infiltrating the skin. Cutaneous lesions from PC patients with strong (ISG^high^ PC, *n* = 6) or weak (ISG^Low^ PC, *n* = 5) I-IFN signatures were examined and compared with those from patients with CLE (*n* = 5), CoV-MPE rashes (*n* = 7), and healthy controls (healthy donors [HD], *n* = 5). We observed a prominent infiltration of CD123^+^ pDCs in both ISG^high^ PC and CLE, but not in ISG^Low^ PC, CoV-MPE rashes, or healthy skin (Fig. 2 A and Fig. S1 A). The CD123^+^ cells in PC lesions were BDCA2^+^, confirming their identity as pDCs (Fig. S1, B and C). CD3^+^ T cells were also more abundant in ISG^high^ PC and CLE, whereas MPO^+^ (myeloperoxidase) neutrophils were present in small numbers in these conditions (Fig. S1 A). In contrast, preferential enrichment in CD163^+^ macrophages was observed in CoV-MPE rashes (Fig. 2 A and Fig. S1 A), consistent with the upregulation of macrophage-related gene transcripts (Fig. S1 D). Macrophages sensing dying endothelial cells via STING have been identified as the principal source of I-IFN and inflammatory cytokines in CoV-MPE rashes (Domizio et al., 2022). Our data therefore suggest that, in chilblains, I-IFN production may be linked to mechanisms other than cell death–induced macrophage activation. Indeed, unlike CoV-MPE rashes, PC display no endothelial cell death (Fig. S1 E). The strong local pDC enrichment in ISG^high^ PC suggests that pDCs may be the source of I-IFN, as these cells are known to be the most potent I-IFN–producing cells in the human body (Gilliet et al., 2008). We found a significant correlation between the numbers of infiltrating CD123^+^ pDCs and the expression of I-IFN response genes (ISG score), whereas no such correlation was found for macrophage numbers (Fig. 2 B). A nuclear p-IRF7 signal was detected in skin-infiltrating CD123^+^ pDCs from ISG^high^ PC, but not in those from ISG^Low^ PC. The pDCs present in ISG^high^ PC were therefore activated and could potentially have been the source of I-IFN (Fig. 2 C). Furthermore, IFN-α staining was detected in CD123^+^ pDCs in two PC lesions (Fig. 2 D; and Fig. S1, F and G), both of which had high ISG scores and a nuclear p-IRF7 signal. Together, these analyses suggest that pDCs may drive the I-IFN response in PC lesions.

### Leukocytes from chilblain patients display enhanced IFN production in response to TLR7 activation

**Figure 3.**
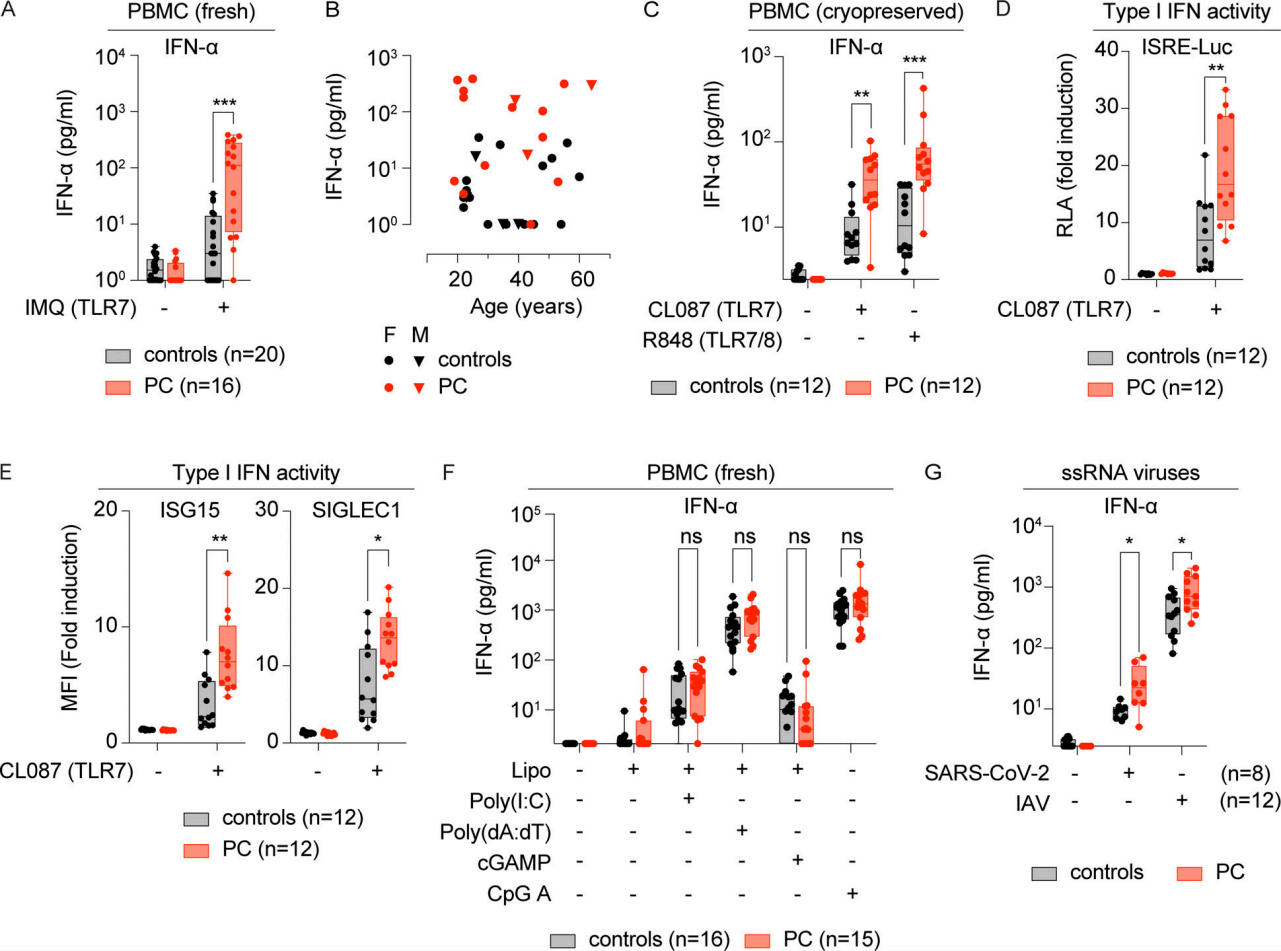
**Enhanced TLR7 responses in patients with PC. (A and B)** PBMCs from chilblain patients (PC, *n* = 16) or HD (controls, *n* = 20) were stimulated with the TLR7-selective agonist IMQ for 24 h. **(A)** Secreted IFN-α was evaluated in a LEGENDplex assay. **(B)** IFN-α production in response to IMQ is shown relative to the age of female (F) and male (M) patients or controls. Freshly isolated PBMCs were used in these experiments. **(C)** PBMCs from chilblain patients (PC, *n* = 12) or HD (controls, *n* = 12) were stimulated with the TLR7-selective agonist CL087 and the dual TLR7/8 agonist R848 for 24 h. Secreted IFN-α was evaluated in a LEGENDplex assay. Cryopreserved PBMCs were used in these experiments. **(D)** ISRE reporter cells (expressing luciferase under the control of an ISRE, THP1-Dual) were treated with supernatants from the CL087-stimulated PBMCs of chilblain patients (PC, *n* = 12) or HD (controls, *n* = 12). Luciferase activity was quantified with a luminometer, and RLA is represented as fold induction over untreated reporter cells. **(E)** THP1-Dual cells were treated with the supernatants of CL087-stimulated PBMCs from chilblain patients (PC, *n* = 12) or HD (controls, *n* = 12). The expression of ISG15 and SIGLEC1 was analyzed by flow cytometry with intracellular staining. Graphs show MFI as fold induction over basal levels in THP1 cells. **(F)** PBMCs from chilblain patients (PC, *n* = 15) or HD (controls, *n* = 16) were stimulated with viral nucleic acid surrogates for 24 h. Secreted IFN-α levels were evaluated in a LEGENDplex assay after stimulation with liposome-encapsulated poly(I:C), cGAMP (STING agonist) or poly(dA:dT) (cytosolic DNA sensors), or naked CpG-A (TLR9 agonist). Freshly isolated PBMCs were used in these experiments. **(G)** PBMCs from chilblain patients (PC) or HD (controls) were stimulated for 24 h with SARS-CoV-2 virus (*n* = 8) or IAV (*n* = 12). Secreted IFN-α was evaluated in a LEGENDplex assay. Cryopreserved PBMCs were used in these experiments. Adjusted P values in A and C–G were determined in Mann–Whitney tests with the Bonferroni correction for multiple testing. ***P < 0.001; **P < 0.01; *P < 0.05; ns = nonsignificant. IMQ, imiquimod; RLA, relative luciferase activity; MFI, mean fluorescence intensity; IAV, influenza A virus.

**Figure S2.**
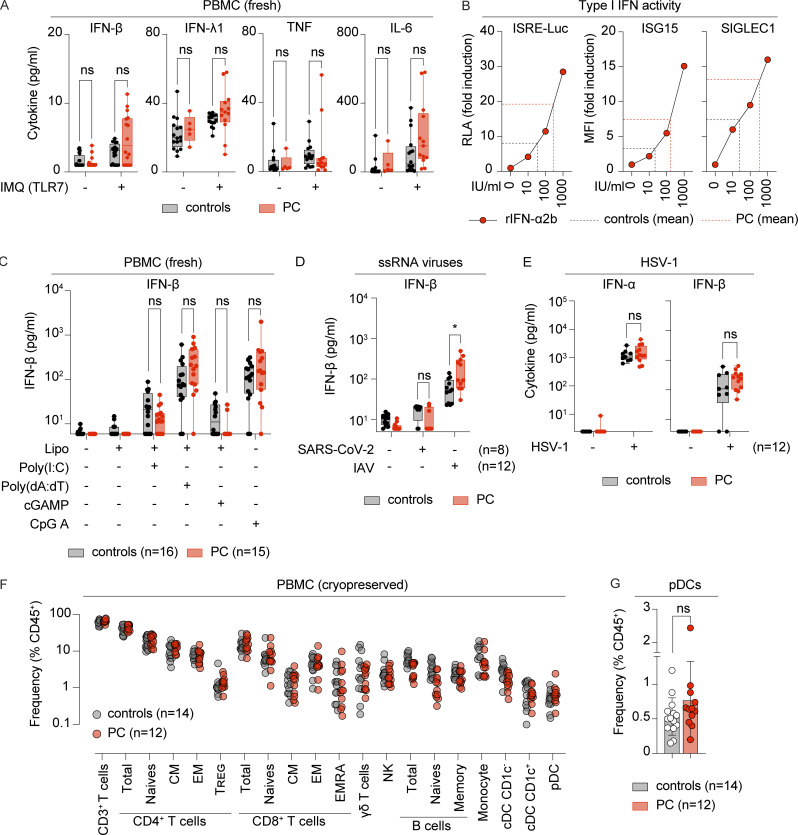
**Related to** Fig. 3 **. (A)** PBMCs from chilblain patients (PC, *n* = 13) or HD (controls, *n* = 16) were stimulated with the TLR7-selective agonist IMQ for 24 h. Secreted cytokines were evaluated in a LEGENDplex assay. Freshly isolated PBMCs were used in these experiments. **(B)** THP1-Dual reporter cells were treated with various doses of recombinant IFN-α (rIFN-α2b), ranging from 10 to 1,000 IU/ml. Left panel: luciferase activity was quantified with a luminometer, and RLA is represented as fold induction over untreated reporter cells. Right panels: the expression of ISG15 and SIGLEC1 was analyzed by flow cytometry with intracellular staining. Graphs show MFI as fold induction over basal levels in THP1 cells. The dashed lines represent the mean type I IFN activity levels for supernatants from the TLR7-stimulated leukocytes of HD (black) (controls, *n* = 12) and chilblain patients (red) (PC, n = 12). **(C)** PBMCs from chilblain patients (PC, *n* = 15) or HD (controls, *n* = 16) were stimulated with viral nucleic acid surrogates for 24 h. Secreted IFN-β levels were evaluated in a LEGENDplex assay after stimulation with liposome-encapsulated poly(I:C), cGAMP (STING agonist) or poly(dA:dT) (cytosolic DNA sensors), or naked CpG-A (TLR9 agonist). Freshly isolated PBMCs were used in these experiments. **(D)** PBMCs from PC patients or controls were stimulated for 24 h with SARS-CoV-2 (*n* = 8) or IAV (*n* = 12). Secreted IFN-β levels were evaluated in a LEGENDplex assay. Cryopreserved PBMCs were used in these experiments. **(E)** PBMCs from PC patients (PC, *n* = 12) or HD (controls, *n* = 12) were stimulated for 24 h with HSV-1. Secreted IFN-α and IFN-β levels were evaluated in a LEGENDplex assay. **(F)** Distribution of lymphoid and myeloid subsets among PBMCs from PC patients (PC, *n* = 12) and HD (controls, *n* = 14) was determined by flow cytometry. Data are represented as percentages of total CD45^+^ cells. CM, central memory T cells; EM, effector memory T cells; EMRA, CD45RA^+^ effector memory T cells; NK, natural killer cells; cDC CD1c^+^, CD1c^+^ conventional dendritic cells; cDC CD1c^−^, CD1c^−^ conventional dendritic cells. **(G)** Frequency of CD123^+^BDCA2^+^ pDCs in PBMCs from PC patients (*n* = 12) and controls (*n* = 14), as analyzed in F, is shown on a separate graph. Bars represent the mean ± SD. The adjusted P values in A, C–E, and G were determined in Mann–Whitney tests with the Bonferroni correction for multiple testing. *P < 0.05; ns = nonsignificant. IMQ, imiquimod; RLA, relative luciferase activity; MFI, mean fluorescence intensity; IAV, influenza A virus.

SARS-CoV-2 is an ssRNA virus sensed by leukocytes via endosomal Toll-like receptor 7 (TLR7) (Asano et al., 2021; Laurent et al., 2022; Onodi et al., 2021; Severa et al., 2021). We hypothesized that the strong I-IFN response observed in PC lesions might be due to an enhanced capacity of the patients’ cells to respond to TLR7 stimulation. We tested this hypothesis by assessing TLR7 responses in a cohort of PC patients (*n* = 16) and a control group of individuals (*n* = 20) of comparable age and sex distribution. Peripheral blood mononuclear cells (PBMCs) collected at least 3 mo after lesion onset were stimulated with imiquimod, a selective TLR7 agonist. Significantly larger amounts of IFN-α were produced by cells from patients with chilblains than by cells from controls (139 versus 9 pg/ml, P = 0.0004) (Fig. 3 A). Enhanced IFN-α production was observed across all ages, for both female and male subjects (Fig. 3 B). The levels of other cytokines (IFN-β, IFN-λ, TNF, and IL-6) remained low in these conditions, although a trend toward slightly higher IFN-β levels in PC was noted (4.8 versus 2.8 pg/ml, P = 0.27) (Fig. S2 A). Stimulation with other TLR7 agonists, such as CL087 and the dual TLR7/8 agonist R848, also resulted in higher levels of IFN-α production in cryopreserved PBMCs from PC patients than in those of controls (Fig. 3 C). We evaluated the biological potency of the I-IFNs produced, by assessing their activity in a luciferase-based IFN-stimulated response element (ISRE) reporter assay (Fig. 3 D) and ISG induction at the protein level (Fig. 3 E). In both assays, supernatants from the TLR7-stimulated leukocytes of patients displayed robust ISRE activation and ISG induction, corresponding to I-IFN activity levels of >100 IU/ml (Fig. S2 B), significantly higher than those for controls (Fig. 3, D and E). We then investigated whether the increase in IFN-α production was specific to the TLR7 pathway, by stimulating leukocytes with ligands for other nucleic acid sensors, including ligands for the RNA sensors TLR3, RIG-I, and MDA-5 (poly(I:C)), the endosomal DNA sensor TLR9 CpG oligodeoxynucleotide type A (CpG-A), and cyclic GMP-AMP synthase (cGAS)/STING-dependent and cGAS/STING-independent cytosolic DNA sensors (cyclic GMP-AMP [cGAMP] and poly(dA:dT)). The activation of nucleic acid sensors other than TLR7 did not induce enhanced IFN-α (Fig. 3 F) or IFN-β (Fig. S2 C) production in patients with PC relative to controls. Overall, our findings suggest that leukocytes from patients with PC are selectively hyperresponsive to TLR7 activation.

### Leukocytes from chilblain patients display enhanced IFN production upon exposure to SARS-CoV-2

We then investigated whether the TLR7 hyperresponsiveness observed in the blood of the patients was associated with an enhanced ability of the patient leukocytes to produce I-IFNs upon exposure to viruses. PBMCs from PC patients or from controls were incubated with SARS-CoV-2 (isolate NY-RU-NY1/2020, ancestral lineage) or another ssRNA virus, influenza A virus (H1N1), which is also known to activate TLR7 (Di Domizio et al., 2009). Exposure to SARS-CoV-2 resulted in significantly higher levels of IFN-α production in cells from PC patients than in cells from controls (29 versus 9.5 pg/ml, P = 0.03) (Fig. 3 G). Enhanced I-IFN production was also observed in response to influenza A virus (Fig. 3 G and Fig. S2 D), indicating that I-IFN overproduction is not restricted to SARS-CoV-2 exposure and may also be triggered by exposure to other TLR7-activating ssRNA viruses. Conversely, the production of IFN-α and IFN-β following stimulation with herpes simplex virus 1 (HSV-1), a DNA virus that does not activate TLR7, was similar in the cells of PC patients and controls (Fig. S2 E). These findings indicate that not all viral stimuli can trigger the overproduction of I-IFNs by leukocytes from PC patients and that this overproduction displays a degree of specificity to TLR7 activation. Thus, patients with PC have enhanced TLR7 responsiveness associated with an overproduction of IFN-α upon exposure to ssRNA viruses.

### Enhanced TLR7-mediated I-IFN production by pDCs in chilblain patients

**Figure 4.**
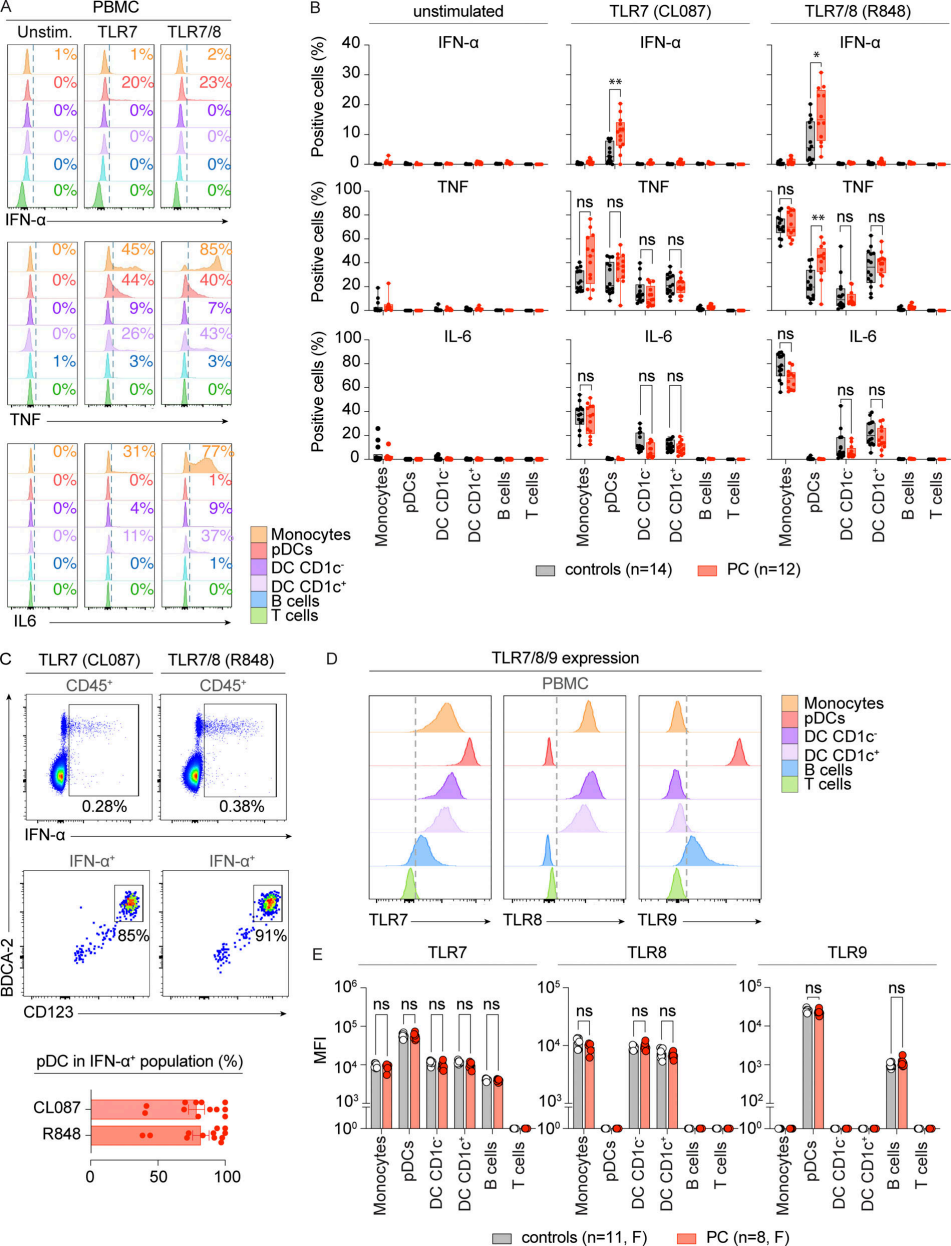
**Enhanced TLR7-mediated I-IFN production by pDCs in chilblain patients. (A)** Intracellular levels of IFN-α (upper panel), TNF (middle panel), and IL-6 (lower panel) in leukocyte subsets were evaluated by flow cytometry after the stimulation of PBMCs for 5 h with the TLR7-selective agonist CL087 and the dual TLR7/8 agonist R848 in the presence of brefeldin A. The subsets studied were as follows: CD14^+^ monocytes (orange), CD123^+^BDCA2^+^ pDCs (red), CD1c^+^ conventional dendritic cells (DC CD1c^+^) (purple), CD1c^−^ conventional dendritic cells (DC CD1c^−^) (light purple), CD19^+^ B cells (blue), CD3^+^ T cells (green). Representative FACS plots from a single patient are shown. The gating strategy is shown in Fig. S3 A. **(B)** Intracellular levels of IFN-α (upper panel), TNF (middle panel), and IL-6 (lower panel) in leukocyte subsets were evaluated by flow cytometry following the stimulation of PBMCs from PC patients (PC, *n* = 12) or HD (controls, *n* = 12), as described in panel A. Graphs represent the percentage of cytokine-positive cells. **(C)** pDCs are the primary source of IFN-α produced in response to stimulation with TLR7 agonists. The upper panel shows the expression of IFN-α by total CD45^+^ PBMCs stimulated with CL087 and R848. The middle panel shows the expression of pDC markers, CD123 and BDCA2, by IFN-α–producing cells. The lower graph shows the percentage of CD123^+^BDCA2^+^ pDCs among IFN-α–producing cells from the PBMCs of PC patients (*n* = 12) stimulated with CL087 and R848. **(D and E)** Intracellular levels of TLR7 (left panel), TLR8 (middle panel), and TLR9 (right panel) in leukocyte subsets were evaluated by flow cytometry on PBMCs. The histograms in D show representative data from a single patient. The graphs in E represent the MFI in PBMCs from female PC patients (PC, *n* = 8) or healthy female donors (controls, *n* = 11). Cryopreserved PBMCs were used in A–E. The adjusted P values in B and E were obtained in Mann–Whitney tests with the Bonferroni correction for multiple testing for non-normally distributed datasets, or in Student’s *t* tests with the Bonferroni correction for multiple testing for normally distributed datasets. Normality was assessed with Shapiro–Wilk and Kolmogorov–Smirnov tests. **P < 0.01; *P < 0.05; ns = nonsignificant; MFI, mean fluorescence intensity.

**Figure S3.**
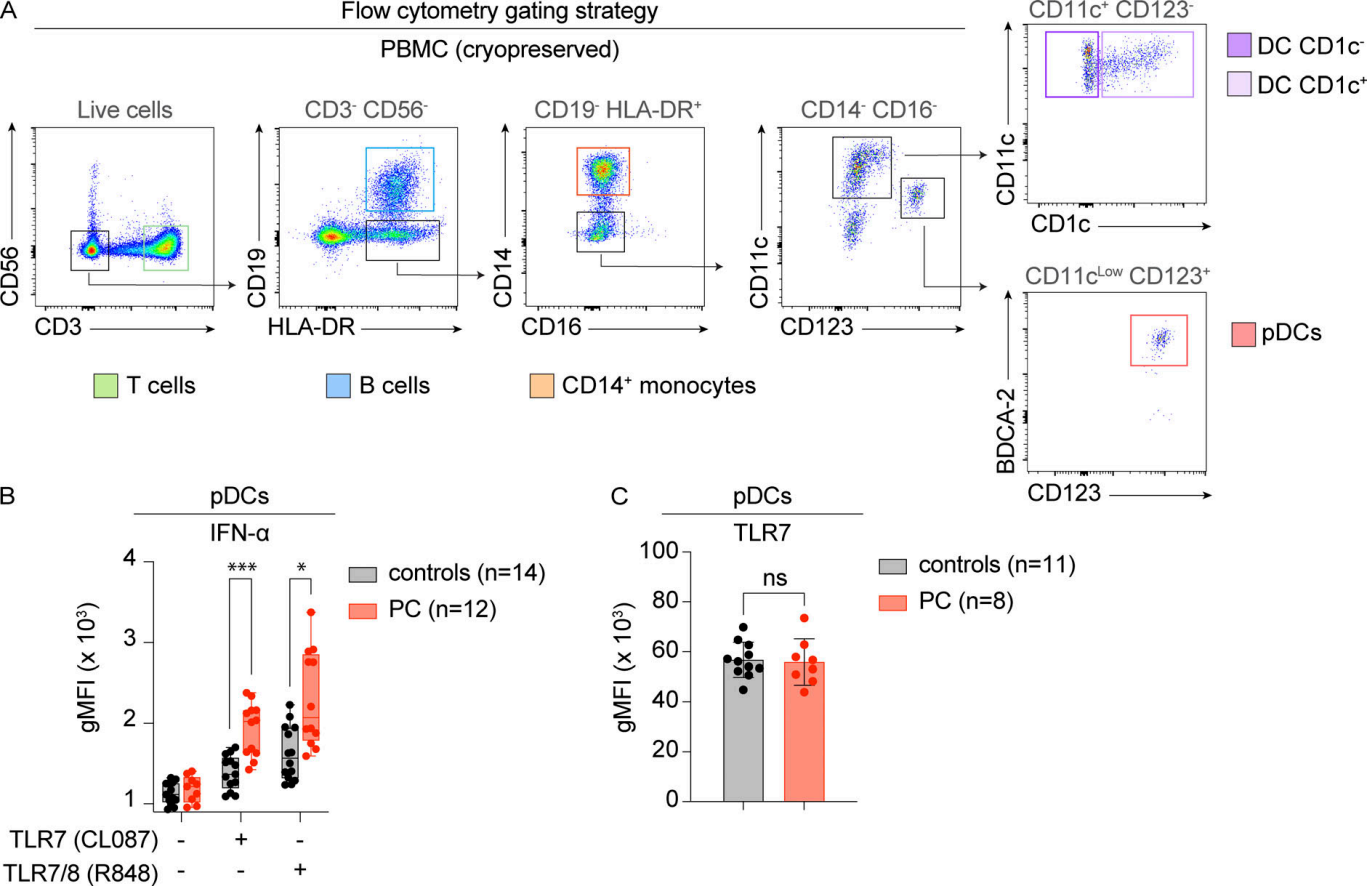
**Related to** Fig. 4 **. (A)** Flow cytometry gating strategy for CD14^+^ monocytes (orange), CD123^+^BDCA2^+^ pDCs (red), CD1c^+^ conventional dendritic cells (DC CD1c^+^) (purple), CD1c^−^ conventional dendritic cells (DC CD1c^−^) (light purple), CD19^+^ B cells (blue), and CD3^+^ T cells (green). Related to Fig. 4, A, B, D, and E. **(B)** Intracellular levels of IFN-α in CD123^+^BDCA2^+^ pDCs were evaluated by flow cytometry after stimulating PBMCs from PC patients (PC, *n* = 12) or HD (controls, *n* = 12) for 5 h with the TLR7-selective agonist CL087 and the dual TLR7/8 agonist R848 in the presence of brefeldin A, as described in Fig. 4 B. The graphs represent the gMFI. **(C)** Intracellular levels of TLR7 in CD123^+^BDCA2^+^ pDCs were evaluated by flow cytometry in PBMCs from female PC patients (PC, *n* = 8) or healthy female donors (controls, *n* = 11). The graphs represent the geometric mean fluorescence intensity (gMFI). Adjusted P values were determined in Mann–Whitney tests with Bonferroni correction in B (non-normally distributed datasets), or Student’s *t* tests with the Bonferroni correction for multiple testing in C (normally distributed datasets). Normality was assessed in Shapiro–Wilk and Kolmogorov–Smirnov tests. ***P < 0.001; *P < 0.05; ns = nonsignificant.

We then performed flow cytometry–based immunophenotyping to analyze the cellular composition of peripheral blood from PC patients and controls. The overall distribution of lymphoid and myeloid subsets (Fig. S2 F), including pDCs (Fig. S2 G), was normal, suggesting that the increase in I-IFN production might be due to cell-intrinsic mechanisms rather than changes to the cellular composition of peripheral blood. We evaluated TLR7 responses in different leukocyte subsets by analyzing the intracellular production of IFN-α, TNF, and IL-6 by flow cytometry following the stimulation of PBMCs with the TLR7-selective agonist CL087 and the dual TLR7/8 agonist R848 (Fig. 4 A and Fig. S3 A). IFN-α production was detected exclusively in CD123^+^BDCA2^+^ pDCs following stimulation with either of the ligands tested, whereas TNF and IL-6 were produced predominantly by CD14^+^ monocytes, particularly in response to stimulation with R848 (Fig. 4, A and B). Cytokine production by CD19^+^ B cells and CD3^+^ T cells was minimal or absent under these conditions. IFN-α production in response to TLR7 stimulation was significantly greater in pDCs from PC patients than in those from controls (Fig. 4 B and Fig. S3 B). In addition, pDCs displayed a significant increase in TNF production in response to R848 stimulation (Fig. 4 B). More than 80% of the IFN-α–producing cells were CD123^+^BDCA2^+^ pDCs (Fig. 4 C), indicating that pDCs are the primary source of IFN-α in TLR7-stimulated PBMCs from PC patients. Together, these findings demonstrate that pDCs from PC patients are hyperresponsive to TLR7 activation and account for the enhanced IFN-α production observed in this context.

### TLR7 levels in pDCs from chilblain patients

We then investigated whether the TLR7 hyperresponsiveness in PC patients could be attributed to higher levels of TLR7 expression levels. We performed flow cytometry analyses to assess the intracellular expression of the endosomal Toll-like receptors TLR7, TLR8, and TLR9 in PBMCs from PC patients and controls (Fig. 4, D and E). TLR7 and TLR8, encoded by adjacent X-linked genes, have been reported to escape X-chromosome inactivation, resulting in higher levels of these receptors in leukocytes from female subjects than in those from male subjects (Souyris et al., 2018; Youness et al., 2023). We avoided confounding effects due to sex-specific differences in TLR7 expression by focusing our analysis exclusively on female patients and controls. We confirmed the expected distribution of the three TLRs across cell types (Fig. 4 D). Importantly, TLR7 levels in pDCs were similar in female PC patients and controls (Fig. 4 E and Fig. S3 C). We found no overall differences between the two groups in terms of the levels of TLR7, TLR8, and TLR9 expressed in the various leukocyte subsets (Fig. 4 E). These findings indicate that enhanced TLR7 responsiveness in PC patients is not associated with any evident increase in TLR7 expression. Together, our results indicate that pDCs from chilblain patients display cell-intrinsic hyperresponsiveness to TLR7 that is independent of changes in TLR7 expression.

### Concluding remarks

Mouse and human pDCs are dendritic cells specialized in nucleic acid sensing and high I-IFN production (Reizis, 2019). Early I-IFN production by mouse pDCs is essential for the control of a limited number of viral infections (Ngo et al., 2024, *Preprint*; Wang et al., 2006), including coronaviruses (Cervantes-Barragan et al., 2007). In humans, the first clear illustration of pDC’s protective role in antiviral immunity was provided by the study of patients with severe COVID-19 pneumonia caused by X-linked recessive TLR7 deficiency (reviewed in Zhang et al. [2022]). This discovery demonstrated that intact ssRNA sensing and I-IFN production by human pDCs are essential for SARS-CoV-2 control in vivo (Asano et al., 2021), a conclusion further supported by several in vitro and observational studies (Cervantes-Barragan et al., 2021, *Preprint*; Onodi et al., 2021; Smith et al., 2022; Venet et al., 2023). Conversely, excessive, delayed, or mislocalized pDC activity can contribute to tissue damage and autoimmune diseases in both mice and humans (Barrat and Su, 2019; Lande et al., 2007; Laurent et al., 2022; Soni et al., 2020), underscoring the critical importance of the magnitude, timing, and localization of pDC responses (Adams et al., 2024; Crow and Casanova, 2024). In this context, our present findings indicate that enhanced ssRNA detection and I-IFN production by pDCs may underlie both robust antiviral protection against SARS-CoV-2 and the development of delayed, distal tissue damage in patients with PC (COVID toes).

**Figure 5.**
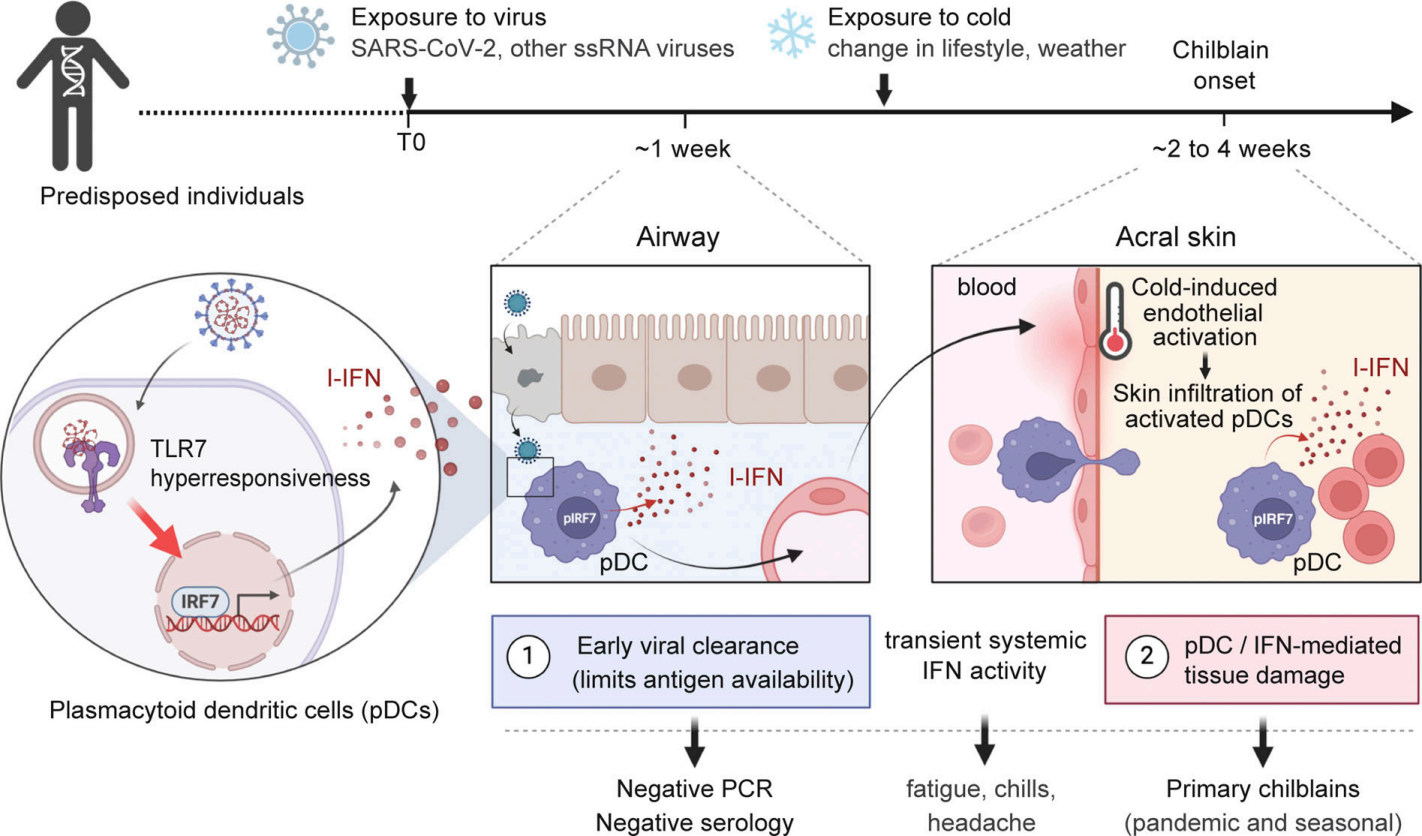
**Proposed model for the pathogenesis of PC.** We propose a two-step model for the immunopathology of PC (COVID toes). In the first step, the pDCs of predisposed individuals with enhanced TLR7 responsiveness produce large amounts of I-IFN in response to exposure to SARS-CoV-2 at the respiratory mucosal site of infection. This may lead to prompt viral clearance, with no adaptive immune markers of infection detectable in most cases (sterilizing innate immunity). In the second step, these patients develop chilblains due to the infiltration of activated pDCs into the skin of the toes, resulting in I-IFN–mediated inflammation. The preferential influx of activated pDCs into acral skin may be linked to the activation of endothelial cells by cold stress resulting from acral coldness or exposure to cold temperatures. Seasonal (or idiopathic) chilblains, which affect otherwise healthy individuals during the winter, may be induced by similar phenomena triggered by winter-specific ssRNA viruses, such as seasonal coronaviruses, respiratory syncytial virus, and influenza A. The figure was generated in BioRender.

We propose a two-step model for the immunopathology of primary chilblains during COVID-19 outbreaks (Fig. 5). In the first step, the pDCs of patients with enhanced TLR7 responsiveness produce large amounts of I-IFN in response to exposure to SARS-CoV-2, potentially leading to prompt viral clearance from the respiratory mucosa. In the second step, these patients develop chilblains due to the infiltration of activated pDCs into the skin of the toes, resulting in I-IFN–mediated inflammation (Aschoff et al., 2020; Battesti et al., 2020; De Greef et al., 2022; Frumholtz et al., 2021; Hubiche et al., 2021a). The preferential influx of activated pDCs into acral skin may be facilitated by the coldness of acral regions (Bergersen and Walløe, 2018). Consistent with this hypothesis, chilblains, including PC, are more likely to occur in the context of cold weather (Neri et al., 2020; Sawires et al., 2022). Acral coldness disrupts local blood flow (Bergersen and Walløe, 2018; Johnson and Kellogg, 2010), resulting in endothelial inflammation and integrin activation, attracting immune cells from the bloodstream to the tissue (Albarrán-Juárez et al., 2018; Nakajima and Mochizuki, 2017). Cold stress also stimulates the migration of immune cells across the endothelium (Awad et al., 2013; Bogert et al., 2020), thereby facilitating the recruitment of activated pDCs to areas of injury (Sozzani et al., 2010). The nature of the ligands sustaining pDC activation within skin lesions remains unclear, but the recent demonstration by Arkin et al. of the presence of sparse SARS-CoV-2 RNA debris in ∼25% of PC lesions despite the absence of mucosal replication or seroconversion provides a compelling potential explanation for the persistence of acral pDC stimulation (Arkin et al., 2024).

Our study does not provide direct evidence of SARS-CoV-2 infection in individuals with PC. However, our findings provide a possible mechanistic explanation for the absence of conventional infection markers in patients with PC. Sterilizing innate immunity, in which the pathogen is cleared exclusively by innate responses without engaging adaptive immunity, has been clearly demonstrated in animal models and involves I-IFNs in particular (Mao et al., 2022; Nice et al., 2015). I-IFNs can play a role in activating B and T cell responses during viral infection (Braun et al., 2002; Seo and Hahm, 2010), but the potent antiviral activity of these molecules can also restrict early viral replication, potentially limiting the availability of the viral antigens required to prime adaptive immune responses. Recent evidence from three prospective SARS-CoV-2–exposed cohorts, including volunteers challenged by direct intranasal inoculation, indicates that SARS-CoV-2 can trigger an early sterilizing I-IFN response that limits viral replication and prevents seroconversion in some exposed individuals (Fenn et al., 2025). In addition, nonseroconversion among individuals recovering from COVID-19 is associated with lower SARS-CoV-2 viral loads in nasopharyngeal swabs (Liu et al., 2021; Weisberg et al., 2021), whereas patients with severe disease or prolonged viral replication develop higher antibody titers (Lucas et al., 2021). Robust I-IFN production in individuals with PC, mediated by TLR7 stimulation and pDCs, may therefore have conferred near-sterilizing innate immunity, limiting antigen availability, thereby accounting for the lack of detectable adaptive immune markers of infection in these patients.

Inherited TLR7 deficiency underlies life-threatening COVID-19 pneumonia and accounts for about 1% of critical cases in men (Asano et al., 2021; Su et al., 2023). Conversely, our findings suggest that enhanced TLR7 responses are associated with protection against SARS-CoV-2. Thus, alterations to the TLR7 pathway may underlie extreme cases at both ends of the disease spectrum, demonstrating the key contribution of TLR7 activity as a major determinant of COVID-19 outcome. The mechanism underlying TLR7 hyperresponsiveness in patients with PC remains to be elucidated. We hypothesize that germline genetic variants of TLR7 pathway genes contribute to the intrinsic ability to mount robust TLR7 responses. The frequent observation of PC in multiple members of the same family (Cordoro et al., 2020; Feder, 2021; Mensa-Vilaró et al., 2022) suggests a genetic predisposition. We are investigating whether affected individuals carry monogenic inborn variants that enhance TLR7-mediated immunity to SARS-CoV-2 or other ssRNA viruses through the enrollment of a dedicated cohort worldwide through the COVID Human Genetic Effort (http://www.covidhge.com). From an evolutionary perspective, leukocytic genes underlying human immunity are under continuous selective pressure to counter infectious threats, resulting in stronger leukocytic responses, albeit at the cost of potentially excessive autoimmunity (Harroud and Hafler, 2023; Kerner et al., 2023). The occurrence of PC, primarily in healthy individuals without chronic autoimmunity, illustrates the delicate balance between enhanced protective innate immunity to an emerging viral threat and the risk of delayed inflammatory damage.

## Materials and methods

### Patient data and samples

Studies on data and samples from patients and healthy volunteers were approved by the institutional review and privacy boards, and the local ethics committees, in accordance with the Helsinki Declaration (2020-02204; CER-VD) for studies using skin and blood samples at Lausanne University Hospital (CHUV) in Lausanne, Switzerland, and protocols JCA-0700 (patients) and JCA-0709 (healthy volunteers) for studies at the Rockefeller University in New York, NY, USA. We enrolled 57 patients with PC (onset between March 2020 and April 2021) in this study. Patients with a history of lupus erythematosus, I-IFN interferonopathies, and related autoinflammatory or autoimmune diseases were excluded. All consecutive patients were recruited. The patients included 38 women (67%), with an age at onset of PC ranging from 9 to 69 years (median of 32 years). Data were collected from patients during consultations in person at the dermatology department (Lausanne University Hospital; 33 of 57, 58%) or teleconsultations (24 of 57, 42%). PCR for SARS-CoV-2 detection was performed on nasopharyngeal swabs collected within 2 wk of chilblain onset, and serological tests for SARS-CoV-2 IgG were performed at least 2 wk after chilblain onset, according to routine laboratory-based diagnostic testing methods. The results of both tests were included in the analysis when available. Participants were classified as having been exposed to SARS-CoV-2 if they reported close contact with COVID-19 patients (laboratory-confirmed or highly suspected cases) within a period of 30 days preceding the onset of chilblains. For skin studies, biopsies were performed on lesions collected from patients with PC at different clinical stages (PC, *n* = 13), patients with diffuse maculopapular eruptions associated with laboratory-confirmed moderate-to-severe COVID-19 (CoV-MPE, *n* = 7), or patients with histologically confirmed CLE (*n* = 6), plaque-type psoriasis (*n* = 7), atopic dermatitis (*n* = 7), or lichen planus (*n* = 7). Control skin samples included biopsy specimens of nonlesional skin from HD (*n* = 7). For leukocyte stimulation assays, whole blood was collected from patients with chilblains (*n* = 16) and controls matched for age and sex (*n* = 20). The individuals in the control group reported mild COVID-19 and no history of severe viral infections, cancer, or autoimmune or autoinflammatory diseases. Informed consent was obtained before the collection of biopsy tissues and blood samples. None of the subjects dropped out of the study, and all samples were analyzed. Randomization was not applicable, and no blinding was performed. Demographic data for the study subjects, including age and sex, are provided in Table S1.

### Immunofluorescence analysis

Formalin-fixed paraffin-embedded skin blocks were sliced into 6-μm sections and mounted on slides. The paraffin was removed, the slides were rehydrated, and heat-induced epitope retrieval and permeabilization with 0.01% Triton in PBS were performed. The sections were stained by overnight incubation at 4°C with primary antibodies in Dako antibody diluent (Cat. S202230-2; Agilent). They were then stained by incubation with fluorescently labeled secondary antibodies for 30 min at room temperature, and the slides were mounted in Fluoroshield mounting agent with DAPI (Sigma-Aldrich). Images were acquired with a Zeiss LSM 700 confocal microscope. For cell quantification, slides were digitalized with a PANNORAMIC 250 Flash digital scanner (3DHISTECH Ltd.) and cell types were quantified with the Measurement tools of SlideViewer 2.5b software (RRID:SCR_024885). For phospho-IRF7 fluorescence quantification, highly infiltrated dermal areas of 3 × 0.136 mm^2^ were analyzed with ImageJ 1.53c software (RRID:SCR_003070) to measure the raw integrated density for pDCs and other cells. The following primary antibodies were used in the study: mouse anti-human CD163 (clone 10D6, Cat. Mob460; DIAGNOSTIC BIOSYSTEMS, dilution 1:50), mouse anti-human CD123 (clone 7G3, Cat. 554527, AB_395455; BD Biosciences, dilution 1:100), rabbit anti-human myeloperoxidase (Cat. A0398, AB_2335676; Agilent, dilution 1:1,000), rabbit anti-human CD3 (clone 2GV6, Cat. 790-4341, AB_2335978; Ventana Medical Systems), mouse anti-human CD31 (JC70A, Cat. M0823, AB_2114471; Agilent, dilution 1:100), rabbit anti-human interferon α 2 antibody (EPR19074, Cat. ab196221, AB_3094694; Abcam, dilution 1:100), rabbit anti-human cleaved caspase-3 (Asp175) (Cat. 9661, AB_2341188; Cell Signaling Technology, dilution 1:200), rabbit anti-human phospho-IRF-7 (Ser471/472) (Cat. 5184, AB_10621425; Cell Signaling Technology, dilution 1:100), and rabbit anti-human CLEC4C (BDCA-2) antibody (EPR21985, Cat. ab239078, AB_2943161; Abcam, dilution 1:500). The secondary antibodies used were donkey anti-rabbit IgG (H+L) Alexa Fluor 546 (Cat. A10040, AB_2534016; Thermo Fisher Scientific, dilution 1:500), goat anti-rabbit IgG (H+L) Alexa Fluor 488 (Cat. A-11008, AB_143165; Thermo Fisher Scientific, dilution 1:500), and goat anti-mouse IgG (H+L) Alexa Fluor 488 (Cat. A-11001, AB_2534069; Thermo Fisher Scientific, dilution 1:500).

### RNA extraction

We collected 4-mm-diameter full-thickness skin fragments by punch biopsy. These fragments were immediately snap-frozen in liquid nitrogen and stored at −80°C until processing. RNA was extracted from tissues by the TRIzol/chloroform method, with a tissue homogenizer (Thermo Fisher Scientific). A_260_/A_280_ values were ≥1.7 for all the RNA preparations obtained. RNA quality was determined using a fragment analyzer (Agilent).

### NanoString analysis

The expression levels of 600 immune genes were determined with the nCounter Human Immunology V2 panel including 20 customized probes (NanoString Technologies) on the nCounter platform (NanoString Technologies). RNA counts were determined by direct probe hybridization with 100 ng RNA per skin sample. Each sample was subjected to quality control checks before inclusion in the analysis. Normalization was performed with nSolver 4.0 (NanoString Technologies) (RRID:SCR_003420) relative to appropriate housekeeping probes selected with the geNorm algorithm (RRID:SCR_006763) implemented in NormqPCR R library48 (RRID:SCR_003388). The final heatmap of differentially expressed genes was generated by gene clustering with the R pheatmap package (RRID:SCR_016418) (for patient clustering: clustering distance = “correlation,” clustering_method = “average”).

### RT-PCR for SARS-CoV-2 on whole-skin extracts

For skin samples, RNA was isolated as described above, and PCR for the detection of SARS-CoV-2 RNA was performed according to the instructions of the TaqMan 2019-nCoV Assay Kit v1 (Applied Biosystems) on QuantStudio 6/7 quantitative PCR instruments (RRID:SCR_020239).

### Isolation and stimulation of PBMCs

Blood from patients (*n* = 16) and healthy controls (*n* = 20) was collected into heparin-containing tubes, and PBMCs were isolated by density gradient centrifugation (Ficoll-Paque) within 4 h of venipuncture. PBMCs from chilblain patients were collected after recovery, at least 3 mo after the onset of symptoms. PBMCs were dispensed into a 96-well U-bottomed plate at a density of 2 × 10^5^ cells per well in 200 μl RPMI 1640 supplemented with 10% fetal bovine serum. Cells were left unstimulated or were incubated with 1 μg/ml imiquimod (Cat#tlrl-imqs; InvivoGen), 5 µM CL-087 (Cayman) and 1 µg/ml R848 (tlrl-r848-5; InvivoGen), 1 μM single-stranded DNA containing unmethylated CpG motifs (CpG-A, Cat#tlrl-2216; InvivoGen), or 1 μg/ml double-stranded RNA polyinosinic-polycytidylic acid (poly(I:C)) (Cat#tlrl-picw; InvivoGen). For the stimulation of intracellular sensors, cells were transfected with nucleic acid surrogates in the presence of Lipofectamine 2000 (Invitrogen). Briefly, 0.3 μl Lipofectamine was mixed with poly(I:C) (Cat#tlrl-picw; InvivoGen), double-stranded DNA poly(deoxyadenylic-deoxythymidylic) acid (poly(dA:dT)) (Cat#tlrl-patn; InvivoGen), or 2′3′-cyclic GMP-AMP (cGAMP) (Cat#tlrl-nacga23; InvivoGen) in 50 μl Opti-MEM. This mixture was incubated for 5 min at room temperature, then dispensed over the surface of 200 μl of cell culture. The final concentration of liposome-encapsulated agonist was 1 μg/ml for poly(I:C), 1 μg/ml for poly(dA:dT), and 5 μg/ml for cGAMP. Depending on the experiment, freshly isolated or cryopreserved PBMCs were used, as indicated in the figure legends. Cryopreserved PBMCs were thawed and allowed to rest in RPMI 1640 supplemented with 10% fetal bovine serum for 2 h before stimulation. All supernatants were collected after 24 h of incubation.

### SARS-CoV-2 virus stocks

The SARS-CoV-2 NYC isolate (isolate NY-RU-NY1/2020, ancestral lineage, GenBank OM345241) was isolated from the saliva of a deidentified patient on July 28, 2020, and propagated in Caco-2 cells (RRID:CVCL_0025) as previously described (Lee et al., 2023). Virus-containing Caco-2 supernatants were clarified, filtered, and titrated on Vero E6 cells (RRID:CVCL_0574). The virus stock used in this study had a titer of 3.4 × 10^6^ plaque-forming units (PFU)/ml.

### Viral stimulations of PBMCs

Cryopreserved PBMCs were dispensed into a 96-well U-bottomed plate at a density of 2 × 10^5^ cells per well in 100 μl RPMI 1640 supplemented with 10% fetal bovine serum and allowed to rest for 2 h before stimulation. PBMCs were stimulated by incubation with the following viruses: SARS-CoV-2 (isolate NY-RU-NY1/2020, ancestral lineage, OM345241) at a multiplicity of infection of 0.1 PFU/cell; influenza A (H1N1) strain A/California/4/2009 at 100 hemagglutination units per milliliter (HAU/ml); and human HSV-1 strain KOS (ATCC VR-1493) at a 1:1,000 dilution. All supernatants were collected after 24 h of incubation. SARS-CoV-2–containing PBMC supernatants were diluted 1:1 in PBS with 0.2% Triton X-100 to inactivate the virus before downstream analysis.

### Cytokine detection

The harvested supernatants of stimulated PBMCs were stored at −20°C until use. The concentrations of cytokines were determined by ELISA for IFN-α (3425-1H-20; Mabtech) and IFN-β (DY814-05; R&D), or by multiplex bead assays with the LEGENDplex Human Antivirus Panel (BioLegend). LEGENDplex reagents (beads and antibodies) were diluted 1:4 in PBS supplemented with 1% bovine serum albumin (BSA). The samples were analyzed by flow cytometry on an Attune NxT flow cytometer (RRID:SCR_019590), with 5,000 beads acquired per sample. Data were analyzed with LEGENDplex Cloud-based Data Analysis Software (Qognit).

### I-IFN activity bioassays

The human monocytic THP1-Dual reporter cell line (thpd-nfis; InvivoGen) (RRID:CVCL_X599) was used to quantify the activity of I-IFNs present in cell supernatants. THP1-Dual cells are derived from THP-1 (RRID:CVCL_0006) cells by the stable integration of a secreted luciferase (Lucia) reporter gene under the control of an ISG54 minimal promoter coupled with five ISREs. Upon exposure to I-IFNs for 24 h, THP1-Dual cells secrete Lucia luciferase, the activity of which can be measured in the supernatant in a luminescence detection assay. The induction of ISGs, such as ISG15 and SIGLEC1, was quantified intracellularly at the protein level by flow cytometry. The response to recombinant human IFN-α and IFN-β was dose-dependent in the 10–1,000 IU/ml range, whereas the TLR7-selective agonist CL-087 elicited no response and IFN-γ induced only a minor response at high doses. The supernatants to be tested were collected from CL-087–stimulated PBMCs. Briefly, PBMCs were dispensed into a 96-well U-bottomed plate at a density of 2 × 10^5^ cells per well in 200 μl of RPMI 1640 supplemented with 10% fetal bovine serum. Cells were either left unstimulated or were stimulated with 5 µM CL-087 (Cayman). After 24 h of incubation, the supernatants were collected, cleared of residual cells by centrifugation, and applied to THP1-Dual cells plated at a density of 1 × 10^5^ cells per well in a 96-well U-bottomed plate. After 24 h, Lucia luciferase activity in the THP1-Dual supernatants was measured with the QUANTI-Luc kit (rep-qlc4lg; InvivoGen) according to the manufacturer’s instructions. The THP1-Dual cell pellets were washed and fixed/permeabilized with the Foxp3 Transcription Factor Staining Buffer Set (eBioscience) according to the manufacturer’s protocol. Permeabilized cells were incubated overnight at 4°C with the following antibodies: ISG15 PE (clone 851701, Cat. IC8044P, RRID:AB_3656663, 1:200 dilution; R&D Systems) and SIGLEC1 APC (clone 7-239, Cat. 130-098-645, RRID:AB_2655547, 1:400 dilution; Miltenyi Biotec), in the presence of Human TruStain FcX (AB_2818986; BioLegend). Stained cells were acquired on an Attune NxT flow cytometer, and data were analyzed with FlowJo software (v10.10.0) (SCR_008520).

### Intracellular cytokine staining

PBMCs were dispensed into a 96-well flat-bottomed plate at a density of 4 × 10^5^ cells per well in 200 μl RPMI 1640 supplemented with 10% fetal bovine serum. The wells were precoated with 2% BSA in PBS to reduce monocyte adhesion. Cells were stimulated with TLR7 agonists CL-087 (5 µM; Cayman) and R848 (tlrl-r848-5, 1 µg/ml; InvivoGen) for 5 h at 37°C in the presence of brefeldin A (TNB-4506-L001, Tonbo, 1X). Following stimulation, cells were harvested, and viability dye staining was performed with ViaKrome 808 (1:2,000 in PBS; Beckman Coulter) for 20 min at room temperature. Cells were then washed and fixed-permeabilized with the Foxp3 Transcription Factor Staining Buffer Set (eBioscience) according to the manufacturer’s instructions. Permeabilized cells were incubated overnight at 4°C with the following antibodies: CD45 Alexa Fluor 350 (clone 2D1, FAB1430U, AB_3646482, 1:200 dilution; R&D Systems); HLA-DR BUV496 (clone L243, 753685, 1:5,000 dilution; BD Biosciences); CD56 BUV737 (clone TULY56, Cat# 367-0566-42, RRID:AB_2895975, 1:400 dilution; Thermo Fisher Scientific); CD11c eFluor 450 (clone 3.9, AB_11218498, 1:400 dilution; Thermo Fisher Scientific); CD123 BV510 (clone 6H6, AB_2562068, 1:800 dilution; BioLegend); CD16 BV570 (clone 3G8, AB_10915988, 1:200 dilution; BioLegend); BDCA-2 BV785 (clone 201A, AB_2572146, 1:800 dilution; BioLegend); CD14 Spark Blue 550 (clone 63D3, AB_2832724, 1:60,000 dilution; BioLegend); CD3 NovaFluor B610-70S (clone SK7, AB_3098363, 1:2,000 dilution; Thermo Fisher Scientific); CD1c PerCP-eFluor 710 (clone L161, AB_10545854, 1:2,000 dilution; Thermo Fisher Scientific); pan-IFN-α PE-Vio 615 (clone REA1013, Cat. 130-116-995, AB_2727805, 1:400 dilution; Miltenyi Biotec); IL-6 PE-Cy7 (clone MQ2-13A5, Cat. 501120, AB_2572042, 1:1,000 dilution; BioLegend); TNF Alexa Fluor 700 (clone Mab11, Cat. 502928, AB_2561315, 1:2,000 dilution; BioLegend); and CD19 APC-Fire810 (clone HIB19, AB_2860770, 1:200 dilution; BioLegend), in the presence of Human TruStain FcX (AB_2818986; BioLegend), True-Stain Monocyte Blocker (Cat. 426102; BioLegend), and CellBlox Plus (C001T06F01; Thermo Fisher Scientific) to minimize nonspecific binding. Cells were acquired on a Cytek Aurora Spectral Flow Cytometer (RRID:SCR_019826), and data were analyzed with FlowJo (v10.10.0) (SCR_008520), with viable cells gated by ViaKrome 808 exclusion and cytokine production analyzed in leukocyte subsets. The gating strategy for leukocyte subsets is shown in Fig. S3 A.

### Analysis of TLR7, TLR8, and TLR9 expression by flow cytometry

PBMCs were washed in PBS and stained with the viability dye ViaKrome 808 (Cat. C36628, Dilution: 1:2,000 in PBS; Beckman Coulter) for 20 min at room temperature. Cells were then fixed-permeabilized with the Foxp3 Transcription Factor Staining Buffer Set (eBioscience) according to the manufacturer’s instructions. Permeabilized cells were incubated overnight at 4°C with the following antibodies: CD45 Alexa Fluor 350 (clone 2D1, FAB1430U, AB_3646482, 1:200 dilution; R&D Systems); HLA-DR BUV496 (clone L243, 753685, 1:5,000 dilution; BD Biosciences); CD56 BUV737 (clone TULY56, RRID:AB_2895975, 1:400 dilution; Thermo Fisher Scientific); CD11c eFluor 450 (clone 3.9, AB_11218498; Thermo Fisher Scientific, 1:400 dilution); CD123 BV510 (clone 6H6, AB_2562068, 1:800 dilution; BioLegend); CD16 BV570 (clone 3G8, AB_10915988, 1:200 dilution; BioLegend); BDCA-2 BV785 (clone 201A, AB_2572146, 1:800 dilution; BioLegend); CD14 Spark Blue 550 (clone 63D3, AB_2832724, 1:60,000 dilution; BioLegend); CD3 NovaFluor B610-70S (clone SK7, AB_3098363, 1:2,000 dilution; Thermo Fisher Scientific); CD1c PerCP-eFluor 710 (clone L161, AB_10545854, 1:2,000 dilution; Thermo Fisher Scientific); TLR7 PE (clone S18024F, AB_2910431, 1:400 dilution; BioLegend); TLR8 APC (clone S16018A, AB_2801050, 1:400 dilution; BioLegend); TLR9 BV421 (clone S16013D, AB_2801039, 1:800 dilution; BioLegend); and CD19 APC-Fire810 (clone HIB19, AB_2860770, 1:200 dilution; BioLegend), in the presence of Human TruStain FcX (AB_2818986; BioLegend), True-Stain Monocyte Blocker (Cat. 426102; BioLegend), and CellBlox Plus (C001T06F01; Thermo Fisher Scientific) to minimize nonspecific binding. Cells were acquired on Cytek Aurora Spectral Flow Cytometer (RRID:SCR_019826), and data were analyzed with FlowJo (v10.10.0) (SCR_008520), with viable cells gated by ViaKrome 808 exclusion and endosomal TLR expression analyzed in leukocyte subsets. The gating strategy for leukocyte subsets is shown in Fig. S3 A.

### Leukocyte immunophenotyping by flow cytometry

PBMCs (0.5 to 1 million) from chilblain patients and controls were stained. Dead cells were labeled with ViaKrome 808 (1:2,000 dilution; Beckman Coulter) in PBS at room temperature for 20 min. Surface staining was then performed by incubating the cells at 4°C for 1 h with the following antibodies: IgD BV480 (clone IA6-2, Cat# 414-9868-41, RRID:AB_2925665, 1:200 dilution; Thermo Fisher Scientific), TCRγδ BUV661 (clone 11F2, Cat# 750019, RRID:AB_2874238, 1:100 dilution; BD Biosciences), CCR6 BV711 (clone G034E3, Cat# 353436, RRID:AB_2629608, 1:100 dilution; BioLegend), CD141 BB515 (clone 1A4, Cat# 565084, RRID:AB_2739058, 1:100 dilution; BD Biosciences), and CD27 NovaFluor Y 590 (clone O323, Cat# H012T02Y02, RRID:AB_2896651, 1:100 dilution; Thermo Fisher Scientific), in the presence of Human TruStain FcX (AB_2818986; BioLegend), True-Stain Monocyte Blocker (Cat. 426102; BioLegend), and CellBlox Plus (C001T06F01; Thermo Fisher Scientific) to minimize nonspecific binding. After washing, cells were fixed and permeabilized with the Foxp3 Transcription Factor Staining Buffer Set (eBioscience) following the manufacturer’s instructions. Permeabilized cells were incubated overnight at 4°C with the following antibodies: CD45RA BUV395 (clone 5H9, Cat# 740315, RRID:AB_2740052, 1:12,000 dilution; BD Biosciences), CCR4 BUV615 (clone 1G1, Cat# 613000, RRID:AB_2870269, 1:100 dilution; BD Biosciences), CD8 BUV805 (clone SK1, Cat# 612889, RRID:AB_2833078, 1:6,000 dilution; BD Biosciences), CCR7 BV750 (clone G043H7, Cat# 353253, RRID:AB_2800944, 1:400 dilution; BioLegend), FoxP3 RB780 (clone 236A/E7, Cat# 569086,, 1:200 dilution; BD Biosciences), CD4 cFluor BYG750 (clone SK3, Cat# SKU R7-20160, 1:6,000 dilution; Cytek), CD127 NovaFluor Red 710 (clone eBioRDR5, Cat# H017T03R04, RRID:AB_2921083, 1:100 dilution; Thermo Fisher Scientific), CD45 Alexa Fluor 350 (clone 2D1, Cat# FAB1430U, RRID:AB_3646482, 1:200 dilution; R&D Systems); HLA-DR BUV496 (clone L243, Cat# 753685, 1:5,000 dilution; BD Biosciences); CD56 BUV737 (clone TULY56, Cat# 367-0566-42, RRID:AB_2895975, 1:400 dilution; Thermo Fisher Scientific); CD11c eFluor 450 (clone 3.9, Cat# 48-0116-41, RRID:AB_11218498, 1:400 dilution; Thermo Fisher Scientific); CD123 BV510 (clone 6H6, Cat# 306022, RRID:AB_2562068, 1:800 dilution; BioLegend); CD16 BV570 (clone 3G8, Cat# 302036, RRID:AB_2632790, 1:200 dilution; BioLegend); BDCA-2 BV785 (clone 201A, Cat# 354221, RRID:AB_2572146, 1:800 dilution; BioLegend); CD14 Spark Blue 550 (clone 63D3, Cat# 367147, RRID:AB_2820021, 1:60,000 dilution; BioLegend); CD3 NovaFluor B610-70S (clone SK7, Cat# H028T03B06, RRID:AB_2910746, 1:2,000 dilution; Thermo Fisher Scientific); CD1c PerCP-eFluor 710 (clone L161, Cat# 46-0015-41, RRID:AB_10545854, 1:2,000 dilution; Thermo Fisher Scientific); and CD19 APC-Fire810 (clone HIB19, Cat# 302272, RRID:AB_2860771, 1:200 dilution; BioLegend). Acquisition was performed with Cytek Aurora Spectral Analyzer (RRID:SCR_019826). Data were analyzed with FlowJo software (v10.10.0) (SCR_008520). Leukocyte subsets were defined as follows: CD8^+^ T cells (CD3^+^ TCRγδ^−^ CD56^−^ CD8^+^), CD4^+^ T cells (CD3^+^ TCRγδ^−^ CD4^+^ FoxP3^−^), regulatory T cells (CD3^+^ CD4^+^ FoxP3^+^ CD127^−^ CD25^+^), γδ T cells (CD3^+^ TCRγδ^+^), natural killer cells (CD3^−^ CD56^+^), B cells (HLA-DR^+^ CD19^+^), classical monocytes (CD14^+^ HLA-DR^+^), conventional dendritic cells (HLA-DR^+^ CD19^−^ CD14^−^ CD11c^+^), and pDCs (BDCA2^+^ CD123^+^ lineage^−^), with T cell subsets further classified as naive, central memory, or effector memory on the basis of CCR7 and CD45RA expression, and B cells classified as naive or memory based on CD27 and IgD expression.

### Statistical analysis

Statistical analyses were performed with GraphPad Prism (RRID:SCR_002798) (v10.4.0), as described in each figure legend. The normality of the distribution of each dataset was assessed in Shapiro–Wilk and Kolmogorov–Smirnov normality tests. For pairwise comparisons of normally distributed datasets, hypotheses were tested in Student’s *t* test (two groups, unpaired two-tailed tests) with the Bonferroni correction for multiple testing. In comparisons of more than two groups with normally distributed datasets, hypotheses were tested in ordinary one-way ANOVA with Tukey’s test for multiple comparisons or Brown–Forsythe Welch’s ANOVA with Dunnett’s test for multiple comparisons, depending on the number of conditions and the experimental setup. For pairwise comparisons of non-normally distributed datasets, statistical significance was determined by Mann–Whitney tests with the Bonferroni correction for multiple testing. For comparisons of more than two groups with non-normally distributed datasets, the Kruskal–Wallis test was performed followed by Dunn’s test for multiple comparisons. P values or adjusted P values below 0.05 were considered significant for all statistical tests.

### Online supplemental material

Fig. S1 is related to Fig. 2. It includes representative confocal microscopy images corresponding to the quantification shown in Fig. 2 A. It also shows BDCA2 and CD123 costaining to confirm the identity of pDCs in PC lesions. In addition, it presents differentially expressed genes in CoV-MPE versus PC ISG^high^. Finally, it provides images and quantifications of cleaved caspase-3 in endothelial cells from skin lesions of CoV-MPE and PC patients. Fig. S2 is related to Fig. 3. It shows the production of additional cytokines (not displayed in Fig. 3) by PBMCs from PC patients or controls stimulated with various agonists, as described in Fig. 3. It also includes THP1-Dual responses to recombinant IFN-α, and PBMC responses from PC patients or controls following HSV-1 stimulation. Finally, it shows the distribution of leukocyte subsets among PBMCs from PC patients and controls. Fig. S3 is related to Fig. 4. It details the flow cytometry gating strategy used to define cell populations shown in Fig. 4, A, B, D, and E. It also shows quantification of intracellular TLR7 expression in pDCs from PC patients and controls. Table S1 summarizes the clinical and demographic characteristics of the PC patients included in this study.

## Supplementary Material

Table S1 shows a summary of epidemiological, clinical, and laboratory data for the PC cases.

## Data Availability

NanoString transcriptomics datasets generated for this study are deposited at GEO data repository under the accession number GSE289528. Reanalyzed NanoString transcriptomics datasets from Seremet et al. (2024) and Domizio et al. (2022) are deposited at GEO data repository under the accession numbers GSE280220 and GSE193068, respectively. All other data are available in the article and its supplementary files.
