# Supplementary material for: Enhanced TLR7-dependent production of type I interferon by pDCs underlies pandemic chilblains
Source: J Exp Med. 2025 Apr 14;222(7):e20231467. doi: 10.1084/jem.20231467 (PMC11995862; doi:10.1084/jem.20231467)
Supplement: Table S1 — shows a summary of epidemiological, clinical, and laboratory data for the PC cases. [file jem_20231467_tables1.docx]

Table S1. Summary of epidemiological, clinical and laboratory data for pandemic chilblains

| Sex |  |  | *N* (%) | | | |
| --- | --- | --- | --- | --- | --- | --- |
|  | Total |  | 57 | | | |
|  | Female |  | 38 (67%) | | | |
|  | Male |  | 19 (33%) | | | |
| Age |  |  | years | | | |
|  | Mean, Median (range) | | 34, 32 (9-69) | | | |
|  |  | Female | 35, 32 (11-69) | | | |
|  |  | Male | 32, 31 (9-64) | | | |
| Chilblain onset | | | *N* (%) | | | |
|  | Total |  | 53 | | | |
|  |  | Mar 2020-Apr 2020 (first wave) | | 25 (47%) | |  |
|  |  | May 2020-Sept 2020 | | 0 (0%) | |  |
|  |  | Oct 2020-Feb 2021 (second wave) | | 21(40%) | |  |
|  |  | Mar 2021-May 2021 (third wave) | | 7 (13%) | |  |
| Relapse | | | *N* (%) | | | |
|  | Total |  | 53 | | | |
|  | Yes | | 8 (15%) | | | |
|  |  | May 2020-Sept 2020 | | | 0 (0%) | |
|  |  | Nov 2020-Feb 2021 (second wave) | | | 7 (87%) | |
|  |  | Mar 2021-May 2021 (third wave) | | | 1 (13%) | |
|  | No |  | 45 (85%) | | | |
| Duration | | | weeks | | | |
|  | Mean, Median (range) | | 9.5, 6 (1-48) | | | |
| Country | | | *N* (%) | | | |
|  | Total |  | 57 | | | |
|  | Switzerland |  | 49 (86%) | | | |
|  | France |  | 7 (12%) | | | |
|  | Luxembourg |  | 1 (2%) | | | |
| Presence of similar lesions in other family members | | | *N* (%) | | | |
|  | Total |  | 57 | | | |
|  | Yes |  | 7 (12%) | | | |
|  | No |  | 50 (88%) | | | |
| Patient recruitment | | | *N* (%) | | | |
|  | Total |  | 57 | | | |
|  | Physical visit (Dermatology Department, CHUV, Lausanne) | | 33 (58%) | | | |
|  | Teleconsultation | | 24 (42%) | | | |
| Exposure to SARS-CoV-2 (before chilblain onset) | | | *N* (%) | | | |
|  | Total |  | 55 | | | |
|  | Yes, contact with a confirmed SARS-CoV-2 case | | 24 (44%) | | | |
|  | Yes, contact with a suspected SARS-CoV-2 case | | 8 (15%) | | | |
|  | No suspicious contact | | 23 (42%) | | | |
| Other symptoms (before/at chilblain onset) | | | *N* (%) | | | |
|  | Total |  | 52 | | | |
|  | Yes | | 17 (33%) | | | |
|  |  | Mild | | | 17 (100%) | |
|  |  | Moderate | | | 0 (0%) | |
|  |  | Severe | | | 0 (0%) | |
|  |  | Headache | | | 9 (53%) | |
|  |  | Fatigue | | | 7 (41%) | |
|  |  | Chills | | | 3 (18%) | |
|  |  | Nasal congestion | | | 3 (18%) | |
|  |  | Fever | | | 1 (6%) | |
|  |  | Pneumonia | | | 0 (0%) | |
|  | No |  | 35 (67%) | | | |
| Nasopharyngeal SARS-CoV-2 PCR (at chilblain onset) | | | *N* (%) | | | |
|  | Tested |  | 36 | | | |
|  | Positive |  | 2 (6%) | | | |
|  | Negative |  | 34 (94%) | | | |
| SARS-CoV-2 IgG serology (after chilblain onset) | | | *N* (%) | | | |
|  | Tested |  | 50 | | | |
|  | Positive |  | 6 (12%) | | | |
|  | Negative |  | 44 (88%) | | | |
| Whole-blood type I IFN signature (at chilblain onset) | | | *N* (%) | | | |
|  | Tested |  | 6 | | | |
|  | Positive |  | 4 (67%) | | | |
|  | Negative |  | 2 (33%) | | | |
| Skin SARS-CoV-2 PCR (lesion, biopsy) | | | *N* (%) | | | |
|  | Tested |  | 9 | | | |
|  | Positive |  | 0 (0%) | | | |
| Skin type I IFN signature (lesion, biopsy) | | | *N* (%) | | | |
|  | Tested |  | 13 | | | |
|  | Positive |  | 7 (54%) | | | |
|  |  | Time from onset: Median (weeks) | | | 4 | |
|  |  | ≤ 2 months | | | 7 (100%) | |
|  |  | > 2 months | | | 0 (0%) | |
|  | Negative |  | 6 (46%) | | | |
|  |  | Time from onset: Median (weeks) | | | 9.5 | |
|  |  | ≤ 2 months | | | 3 (50%) | |
|  |  | > 2 months | | | 3 (50%) | |
